# Enhancement of proinflammatory and procoagulant responses to silica particles by monocyte-endothelial cell interactions

**DOI:** 10.1186/1743-8977-9-36

**Published:** 2012-09-18

**Authors:** Xin Liu, Yang Xue, Tingting Ding, Jiao Sun

**Affiliations:** 1 Shanghai Biomaterials Research & Testing Center, Shanghai Key Laboratory of Stomatology, Ninth People’s Hospital, Shanghai Jiaotong University School of Medicine, No. 427, Ju-men Road, Shanghai, 200023, China

**Keywords:** Endothelial cells, Monocytes, Inflammation, Particles, Cell-cell interaction, Signal transduction

## Abstract

**Background:**

Inorganic particles, such as drug carriers or contrast agents, are often introduced into the vascular system. Many key components of the *in vivo* vascular environment include monocyte-endothelial cell interactions, which are important in the initiation of cardiovascular disease. To better understand the effect of particles on vascular function, the present study explored the direct biological effects of particles on human umbilical vein endothelial cells (HUVECs) and monocytes (THP-1 cells). In addition, the integrated effects and possible mechanism of particle-mediated monocyte-endothelial cell interactions were investigated using a coculture model of HUVECs and THP-1 cells. Fe_3_O_4_ and SiO_2_ particles were chosen as the test materials in the present study.

**Results:**

The cell viability data from an MTS assay showed that exposure to Fe_3_O_4_ or SiO_2_ particles at concentrations of 200 μg/mL and above significantly decreased the cell viability of HUVECs, but no significant loss in viability was observed in the THP-1 cells. TEM images indicated that with the accumulation of SiO_2_ particles in the cells, the size, structure and morphology of the lysosomes significantly changed in HUVECs, whereas the lysosomes of THP-1 cells were not altered. Our results showed that reactive oxygen species (ROS) generation; the production of interleukin (IL)-6, IL-8, monocyte chemoattractant protein 1 (MCP-1), tumor necrosis factor (TNF)-α and IL-1β; and the expression of CD106, CD62E and tissue factor in HUVECs and monocytes were significantly enhanced to a greater degree in the SiO_2_-particle-activated cocultures compared with the individual cell types alone. In contrast, exposure to Fe_3_O_4_ particles had no impact on the activation of monocytes or endothelial cells in monoculture or coculture. Moreover, using treatment with the supernatants of SiO_2_-particle-stimulated monocytes or HUVECs, we found that the enhancement of proinflammatory response by SiO_2_ particles was not mediated by soluble factors but was dependent on the direct contact between monocytes and HUVECs. Furthermore, flow cytometry analysis showed that SiO_2_ particles could markedly increase CD40L expression in HUVECs. Our data also demonstrated that the stimulation of cocultures with SiO_2_ particles strongly enhanced c-Jun NH_2_-terminal kinase (JNK) phosphorylation and NF-κB activation in both HUVECs and THP-1 cells, whereas the phosphorylation of p38 was not affected.

**Conclusions:**

Our data demonstrate that SiO_2_ particles can significantly augment proinflammatory and procoagulant responses through CD40–CD40L-mediated monocyte-endothelial cell interactions via the JNK/NF-κB pathway, which suggests that cooperative interactions between particles, endothelial cells, and monocytes may trigger or exacerbate cardiovascular dysfunction and disease, such as atherosclerosis and thrombosis. These findings also indicate that the monocyte-endothelial cocultures represent a sensitive *in vitro* model system to assess the potential toxicity of particles and provide useful information that may help guide the future design and use of inorganic particles in biomedical applications.

## Background

Due to their excellent mechanical stability, high carrier capacity, easy variation of surface properties and inexpensive synthesis, inorganic nanoparticles have been widely studied in various medical fields, such as drug delivery, the discovery of biomarkers, and molecular diagnostics and gene therapy [1]. Before nanoparticles are used for medical applications, their biological behavior and toxicological properties must be carefully assessed. Thus, it is necessary to understand the interactions of nanoparticles with biological systems.

For many intravenously administered nanoparticle-based drug carriers, the prolonged circulation properties can lead to the controlled release of therapeutic agents in the blood to targeted cells. However, the extended circulation time may increase the duration of the particles’ contact with blood components and endothelium and potentially cause undesirable host responses. Monocytes are among the first immune cells recruited to an invasion site in response to foreign materials. Recently, many studies have focused on nano-immunotoxicity and have found that some inorganic particles (*e.g.,* hydroxyapatite particles, Nano-Co, and quantum dots) can activate monocytes to increase the release of proinflammatory cytokines and reactive oxygen species (ROS) [2-4]. Monocytes are a commonly used *in vitro* model for the innate immune response within a single cell type, but in the case of barrier defense, more complex models are required [5]. The endothelium not only serves as a natural barrier in controlling the passage of particles from the blood into the surrounding tissues but also intricately links to innate immunity. Previous studies have shown that most inorganic particles (*e.g.,* silica, zinc oxide, and alumina particles) can initiate an inflammatory response in endothelial cells (ECs), including the secretion of proinflammatory cytokines and the upregulation of vascular cellular adhesion molecule-1 (VCAM-1), intercellular adhesion molecule-1 (ICAM-1) and E-selectin, which are responsible for monocyte recruitment and adhesion [6-8]. Monocyte-endothelial cell adhesion and interactions have long been recognized for their essential roles in the process of inflammation and thrombosis [9]. However, to date, while the direct effects of particles on ECs and monocytes have been widely discussed, far less effort has been put forth concerning the question of whether the particles can indirectly influence the host immune response through ECs or indirectly induce endothelial cell dysfunction via monocytes. Thus, the functional consequences and precise mechanisms of particle-induced monocyte-endothelial cell interactions must be further investigated. Ongoing applications of engineered nanoparticles in drug delivery systems and the molecular imaging field increase the urgency of such studies. In general, the interactions between monocytes and ECs may be direct, through ligand-receptor interactions, or indirect, through released factors (*e.g*., cytokines, growth factors or ROS). [10,11] Recently, it has been reported that CD40/CD40L-mediated costimulation between monocytes and ECs leads to the induction of inflammatory and adhesive proteins in both cell types [12,13]. Moreover, there is increasing evidence that particles can effectively upregulate CD40 expression in immune cells [14,15]. Thus, it is likely that both soluble factors and costimulatory molecules play critical roles in particle-mediated monocyte-endothelial cell interactions, and further investigations are required to support this hypothesis.

Metal and silica particles (SiO_2_ particles) are among the most promising inorganic particles being developed for target therapy or molecular imaging [16-18]. Thus, Fe_3_O_4_ and SiO_2_ particles were chosen as test materials in the present study. As drug carriers or contrast agents, the distribution of particles into the vascular system appears highly probable. In our previous studies, we have found that SiO_2_ particles could directly induce inflammatory activation in ECs by the NF-κB pathway [8]. Here, considering the complex architecture of the vascular system, we established a coculture model of THP-1 cells (monocytes) and human umbilical vein endothelial cells (HUVECs) to mimic the *in vivo* situation and, for the first time, investigated the integrated effects and possible mechanisms of the interactions between particles, monocytes and ECs. First, we assessed the direct effects of particles on THP-1 cells and HUVECs through the observation of cellular uptake and changes in cell viability. Subsequently, to investigate the functional consequences and molecular mechanisms of particle-mediated monocyte-endothelial cell interactions, we measured ROS levels, the release of proinflammatory cytokines, cellular adhesion molecules (CAMs), procoagulant marker expression, mitogen-activated protein kinases (MAPK), and the NF-κB activation of monocytes and ECs in particles-stimulated mono- and co-cultures. Moreover, to determine the role of soluble factors and cell-to-cell contact in particle-induced monocyte-endothelial cell interactions, we used the supernatant from THP-1 cells that had been stimulated with particles to treat HUVECs and vice versa and then examined the proinflammatory and procoagulant responses. In addition, to investigate the cell-to-cell contact-dependent mechanism, we also measured CD40L and CD40 expression in particle-stimulated THP-1 cells and HUVECs. Our studies provide a better understanding of the impact of nanoparticles on monocyte-endothelial cell interactions, which aids in the design of nanoparticles for various applications, including drug delivery or molecular imaging, especially when the cellular microenvironment near an atherosclerotic plaque site must be considered.

## Results and discussion

**Figure 1:**
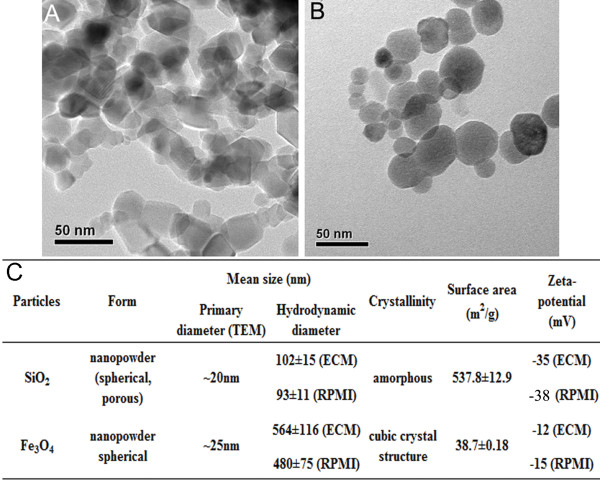
**Characterization and cytotoxicity of the particles. A-B**): TEM analysis of particles. **C**): Particle size, hydrodynamic diameter, surface area and zeta potential.

Prior to investigating the biological effects of particles used in the current studies, the particles were characterized with a transmission electron microscope (TEM), dynamic light scattering (DLS), and nitrogen adsorption-desorption isotherms. The TEM analysis revealed that the primary size of SiO_2_ and Fe_3_O_4_ particles was approximately 20 nm and 25 nm in diameter, respectively, and the shape was near spherical (Figure 1A-B). In aqueous systems, nanoparticles have a tendency to aggregate. Therefore, the secondary particles’ size in aqueous solutions (the hydrodynamic size) might also be an important factor affecting their biological behaviors. As shown in Figure 1C, the hydrodynamic size was 102 nm in EC medium (ECM) and 93 nm in RPMI 1640 medium with 10% FBS for SiO_2_ particles, and 564 nm in ECM and 480 nm in RPMI 1640 medium with 10% FBS for Fe_3_O_4_ particles. Subsequently, the measurement of zeta potential was also used to study the agglomeration and dispersion stability of the colloidal system. The higher the zeta potential, the more likely that the suspension is stable. In general, particle suspensions with an absolute zeta potential value above 30 mV are normally considered stable. Consistent with the DLS measurement results, Fe_3_O_4_ particles had the lowest absolute magnitude of zeta potential (12 mV in ECM and 15 mV in RPMI), followed by SiO_2_ particles (35 mV in ECM and 38 mV in RPMI), indicating that SiO_2_ particles have a lower degree of agglomeration and higher dispersion stability than Fe_3_O_4_ particles in culture media. Most studies have demonstrated that the agglomeration of particles results in a decrease in the associated toxicity [19,20]. However, recent studies have found that agglomerated SiO_2_ particles induce more potent proinflammatory cytokine responses than non-agglomerated particles, indicating that avoiding agglomeration may lead to an underestimation of the possible adverse effects [21]. Thus, it might be more important to conduct a thorough characterization of the agglomeration states than to maintain a single-particle preparation when analyzing the potential health hazard of the particles. In addition, the surface area is also an important physico-chemical parameter of particles. Our Brunauer–Emmett–Teller (BET) data indicated that the SiO_2_ particles have a larger surface area (537.8 m^2^/g) than the Fe_3_O_4_ particles (Figure 1C). The aforementioned characteristics would help us to better analyze the biocompatibility and toxicity properties of the particles. 

### Cytotoxicity of the particles

**Figure 2:**
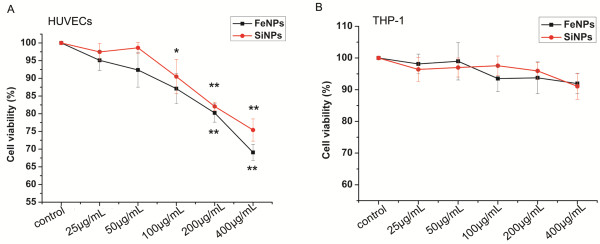
**Cytotoxicity of the particles to HUVECs and THP-1 cells.A**): HUVECs and **B**): THP-1 cells. Cells were exposed to increasing doses of particles for 24 h, and the cytotoxicity was determined by the MTS assay. Normal HUVECs or THP-1 cells without particle treatment served as controls. The results are presented as the mean ± SEM of three independent experiments, each of which was carried out in triplicate. *p < 0.05, **p < 0.01 vs. control. (FeNPs: Fe_3_O_4_ particles; SiNPs: SiO_2_ particles).

In this study, THP-1 cells and HUVECs were used to investigate the biological effects of SiO_2_ or Fe_3_O_4_ particles. Monocytes are involved in the first line of defense in the immune system and protect the body as a scavenger of foreign agents via phagocytosis. As a result, evaluating nanotoxicity using these two cell types can provide a comprehensive immuno-inflammatory nanotoxicity assessment of particles. Generally, the performance of cell viability assays is a basic step in nanotoxicology that demonstrates the cellular response to particles. Herein, the cytotoxicity of SiO_2_ or Fe_3_O_4_ particles in HUVECs and THP-1 cells was measured by the MTS method, a type of mitochondrial succinate dehydrogenase assay. Several previous studies have shown that reagents from the MTT or LDH assays can bind to particles and produce invalid results due to particle/dye interactions or the adsorption of the dye or dye products [22,23]. However, in contrast to MTT or LDH, the MTS indicator dye is water soluble and stable in the culture medium, and it could only minimally interact with the particles. Therefore, we chose to use the MTS assay to assess the cytotoxicity of the particles. In HUVECs, a dose-dependent toxic effect was observed after exposure to SiO_2_ or Fe_3_O_4_ particles at concentrations ranging from 100 to 400 μg/mL. Exposure to SiO_2_ or Fe_3_O_4_ particles at concentrations of 200 μg/mL and above caused significant cytotoxic effects (Figure 2A). The cell types differed in viability; in THP-1 cells, no significant loss of viability was observed at any of the concentrations tested (Figure 2B). 

### Particle uptake

**Figure 3:**
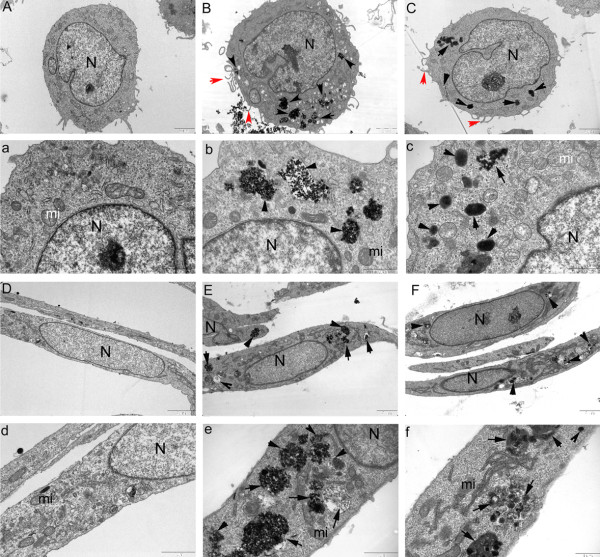
**Uptake of particles by HUVECs and THP-1 cells.** TEM micrographs of cells exposed for 24 h to particles. **A**) and **a**): THP-1 cells without any treatment; **B**) and **b**): THP-1 cells treated with FeNPs; **C**) and **c**) THP-1 cells treated with SiNPs; **D**) and **d**): HUVECs without any treatment; **E**) and **e**): HUVECs treated with FeNPs; **F**) and **f**): HUVECs treated with SiNPs; (**A**-**F**): Overall cell morphology (scale bar: 2 μm). (**a**-**f**): Higher magnification of part of the area in the cells (scale bar: 1 μm). (N: nucleus, mi: mitochondria). Black arrows denote NPs. Red arrows indicate the protrusion of the plasma membrane for phagocytosis. ( FeNPs: Fe3O4 particles; SiNPs: SiO2 particles).

Phagocytosis is mainly conducted by specialized mammalian cells, such as macrophages, monocytes and neutrophils [15,24]. In nonphagocytic cells, there are three major endocytic pathways: macropinocytosis, clathrin-mediated endocytosis, and caveolin-dependent endocytosis [24,25]. The plasma membrane protrusion for cellular uptake is one of the characteristics of phagocytosis [15]. As depicted in Figure 3, our results showed that multiple pseudopodia of plasma membrane were formed for the uptake of the SiO_2_ or Fe_3_O_4_ particles in monocytes but not HUVECs, indicating that SiO_2_ or Fe_3_O_4_ particles might enter into monocytes via phagocytosis and into ECs via other endocytic pathways. Moreover, in both cell types, most particles were observed to sequester in vesicles and lysosomes; however, there was no evidence of particles entering nuclei and mitochondria, suggesting that the final destination of transported SiO_2_ or Fe_3_O_4_ particles is the lysosomes. The results are consistent with other studies that showed that particles are preferentially localized in the lysosomes of HeLa cells and human breast-cancer cells [26,27]. Notably, in THP-1 cells, despite the fact that the lysosomes engulfed large numbers of particles, the morphology did not change significantly, and the structure remained homogeneous. In contrast, with the accumulation of particles in HUVECs, the size of the lysosomes significantly increased, while the structure and morphology became irregular and indicative of cellular perturbation by events within lysosomes. Lysosomal perturbation might be a major mechanism for particle cytotoxicity and explain why HUVECs are more susceptible to particles than monocytes. Because the subsequent experiments required functioning and metabolically active cells, a low dose (100 μg/mL) that did not significantly affect the viability of monocytes or ECs was used in this study. 

### Monocytes amplify particle-induced endothelial cell inflammatory responses

**Figure 4:**
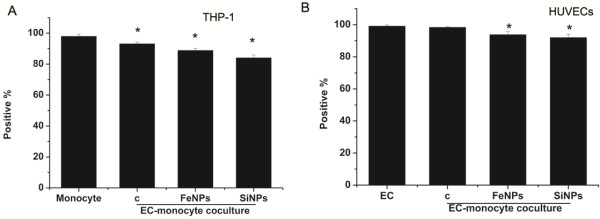
**The purity of HUVECs and THP-1 cells from cocultures.** The bar graph shows **A**): CD11a positive THP-1 cells and **B**): vWF positive HUVECs. Data represent the means ± SEM; n = 3. *p < 0.05 vs. control. (c: control, FeNPs: Fe_3_O_4_ particles; SiNPs: SiO_2_ particles, EC: endothelial cells).

**Figure 5:**
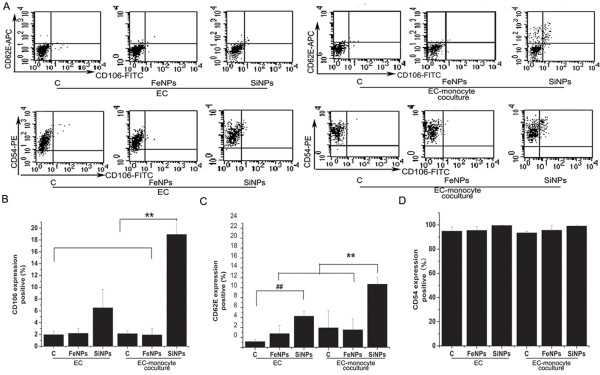
**Expression of cell adhesion molecules induced by particles in HUVECs in mono- and co-cultures. A**): Dot-plot of flow cytometry analysis showing the expressions of CD54, CD106, and CD62E on HUVECs in mono-and co-cultures exposed to particles for 24 h. **B**): Bar graph shows CD106 expression, **C**): CD62E expression, and **D**) CD54 expression. Untreated HUVECs served as the negative control. Data represent the means ± SEM; n = 3. ##, **p < 0.01, a significant difference between compared groups. (c: control, FeNPs: Fe_3_O_4_ particles, SiNPs: SiO_2_ particles, EC: endothelial cells).

**Figure 6:**
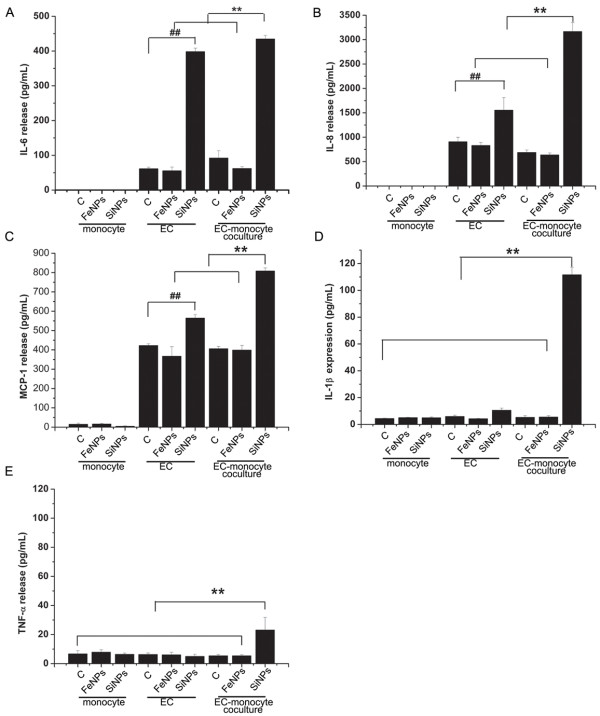
**Cytokine production induced by particles in mono- and co-cultures. A**): IL-6 release; **B**): IL-8 release; **C**): MCP-1 release; D): IL-1β release; E): TNF-α release. Untreated HUVECs or THP-1 cells served as negative controls. Data represent the mean ± SD, n = 3. ## p < 0.01, **p < 0.01, a significant difference between compared groups. (c: control, FeNPs: Fe_3_O_4_ particles; SiNPs: SiO_2_ particles, EC: endothelial cells).

Previous studies have examined the effects of particles on both HUVEC and THP-1 cells in monocultures, whereas the effects of particles on cell–cell interactions have not been investigated in detail. In the present study, we established a coculture model that permits direct communication and the interaction of monocytes with ECs to assess the potential proinflammatory and prothrombotic risks of SiO_2_ or Fe_3_O_4_ particles. Prior to investigating monocyte-EC interactions, the purity of HUVECs and THP-1 cells was assessed by flow cytometry. Our data showed that the purity of monocytes or HUVECs isolated from a silica particle-treated coculture was slightly lower than that of monocytes or HUVECs isolated from an untreated coculture, suggesting that the particles may elicit the attachment of monocytes to HUVECs. However, the low level of contamination with the other cell type after separation from cocultures (<10%) would not likely affect the measurements (Figure 4). Endothelial cell activation, a proinflammatory and procoagulant state of the ECs, is characterized by the upregulation of adhesion molecule expression, including VCAM-1 (CD106), ICAM-1 (CD54) and E-selectin (CD62E) [28]. Thus, in our initial experiments, we used particles to stimulate the coculture system and examined the expression of CD54, CD106 and CD62E in HUVECs. As illustrated in Figure 5, untreated quiescent HUVECs express constitutive levels of surface CD54 but no detectable CD106 or CD62E. In a monoculture of HUVECs, only CD62E expression was slightly upregulated by SiO_2_ particles. It was unexpected that when HUVECs were cocultured with THP-1 cells, both CD106 and CD62E expression were markedly increased upon exposure to the SiO_2_ particles compared with the HUVEC monoculture stimulated with SiO_2_ particles, while no change in CD54 expression was observed. Both CD106 and CD62E play an important role in recruiting monocytes and mediating monocyte rolling and adhesion to the internal surface of the blood vessel. In addition to upregulating adhesion molecules, ECs react to stimuli by secreting various proinflammatory cytokines and chemokines, including interleukin (IL)-8, IL-6 and monocyte chemoattractant protein (MCP)-1. Our results also indicate that SiO_2_ particles significantly upregulated the production of IL-6, IL-8, and MCP-1 in a monocyte/HUVEC co-culture system, compared with HUVECs alone cultured with SiO_2_ particles. Because THP-1 cells alone cultured with SiO_2_ particles did not elicit detectable IL-6, IL-8, and MCP-1 responses (Figures 6A-C), the results indicate that SiO_2_-particle-induced cell–cell interactions led to an increased IL-6, IL-8, and MCP-1 production by ECs. IL-6 has been shown to increase smooth muscle cell proliferation and migration and may promote atherosclerotic lesions and plaque vulnerability through the stimulation of acute-phase protein synthesis [29,30]. In addition, both MCP-1 and IL-8 also contribute to arteriosclerotic lesion formation and trigger firm adhesion of monocytes to the EC layer [31]. Previous studies have reported that iron oxide particles’ challenge to ECs upregulates the expression of cell adhesion molecules and cytokines, promotes monocyte adhesion to ECs and contributes to the initial development of atherosclerosis [32]. In contrast, other studies have demonstrated that Fe_2_O_3_ NPs do not provoke an inflammatory response in endothelial cells [6]. In our studies, we found that Fe_3_O_4_ particles have no significant effect on endothelial cell adhesion molecule expression or cytokine release in a monoculture or cocultures. Our present data indicate the presence of a positive feedback loop that enables the adhesion of monocytes to ECs, thus contributing to the proinflammatory responses of vascular endothelium upon exposure to SiO_2_ particles. 

### EC-mediated proinflammatory and procoagulant activation of monocytes in response to SiO_2_ particles

Having shown that monocyte-endothelial cell interactions can enhance the endothelial inflammatory response to SiO_2_ particles, we analyzed monocyte activation to test whether this phenomenon is restricted to ECs. TNF-α and IL-1β were used as markers of monocyte activation. Notably, neither HUVECs nor monocytes produced TNF-α or IL-1β with SiO_2_-particle treatment. However, a massive amplification of TNF-α (~5-fold) and IL-1β (~10-fold) secretion was observed in response to SiO_2_ particles, but not Fe_3_O_4_ particles, in the monocyte/EC coculture compared with THP-1 cells or HUVECs alone stimulated with particles, suggesting that monocytes/ECs in coculture are more responsive to SiO_2_ particles than either cell type alone (Figures 6D-E). Many previous studies have shown that cocultures of multiple cell types have an increased sensitivity to microparticles and release more proinflammatory cytokines than one cell type alone [33-35]. It has been reported that mesoporous SiO_2_-particles hardly induce proinflammatory cytokines, such as TNF-α and IL-1β, in macrophages [36]. However, an increased release of proinflammatory cytokines (TNF-α and IL-1β) in blood after injection with SiO_2_ particles has previously been observed *in vivo*[37]. Taken together with our results, it is likely that SiO_2_ particles can indirectly activate monocytes through the stimulation of ECs.

**Figure 7:**
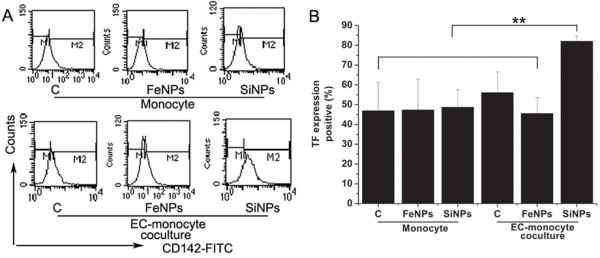
**The effect of particles on tissue factor (TF) expression in THP-1 cells in mono- and co-cultures. A**): TF expression is depicted by a histogram of flow cytometry analysis. **B**): The bar graph shows the percentage of TF positive cells. Data represent the means ± SEM; n = 3. **p < 0.01, a significant difference between compared groups. (c: control, FeNPs: Fe_3_O_4_ particles; SiNPs: SiO_2_ particles, EC: endothelial cells).

An important mechanism whereby vascular inflammation can contribute to thrombosis is through the up-regulation of tissue factor (TF) expression [38], which results in the activation of the extrinsic blood coagulation cascade. Monocytes have been recognized as the main cell type that can be induced to synthesize TF *de novo*. Therefore, we subsequently investigated the expression of TF by THP-1 cells in monoculture and cocultures exposed to particles. Figure 7 shows that THP-1 cells alone did not exhibit any increase in the expression of TF after exposure to SiO_2_ or Fe_3_O_4_ particles. Another group has also reported that synthetic amorphous SiO_2_ particles had virtually no effect on TF gene transcription in monocytes [39]. However, in the present study, THP-1 cells cocultured with HUVECs exhibited a significant increase in TF expression in response to SiO_2_ particles, while Fe_3_O_4_ particles did not induce TF expression in THP-1 cells in cocultures. Data generated by these experiments suggest that even though SiO_2_ particles have no proinflammatory or procoagulant activity on ECs or monocytes alone, their proinflammatory and procoagulant potential can be dramatically induced and augmented by particle-induced monocyte-endothelial cell interactions. 

### Soluble factors and cell-to-cell contact-dependent mechanisms

**Figure 8:**
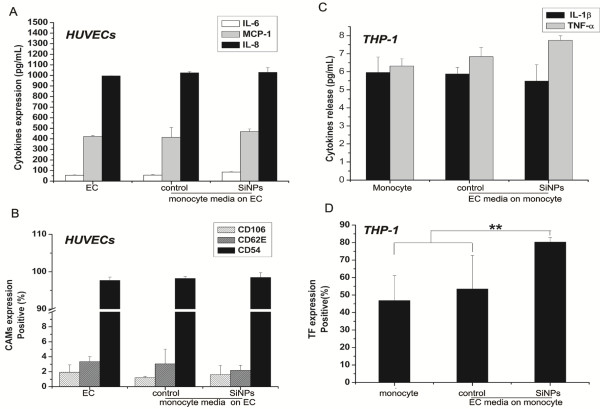
**The expression of cytokines, CAMs and TF in HUVECs and THP-1 cells treated with the supernatants of SiO**_**2**_**-particle-stimulated cells. A**): IL-6, IL-8 and MCP-1 release; **B**): Expression of cell adhesion molecules (CAMs) in HUVECs treated by the supernatants of SiO_2_-particle-stimulated THP-1 cells; **C**): TNF-α and IL-1β release; **D**) TF expression in THP-1 cells treated by the supernatants of SiO_2_-particle-stimulated HUVECs. Data represent the means ± SEM; n = 3. **p < 0.01, a significant difference between compared groups. (SiNPs: SiO_2_ particles, EC: endothelial cells).

In the next step, we investigated whether soluble factors from SiO_2_-particle-stimulated monocytes are responsible for EC activation. Thus, cell-free supernatants of monocytes activated with SiO_2_-particles were used to stimulate HUVECs. Interestingly, although cocultures activated with SiO_2_ particles resulted in the dramatic enhancement of EC activation, the supernatant of SiO_2_-particle-stimulated monocytes did not upregulate CAMs expression or the release of cytokines in HUVECs (Figures 8A-B), suggesting that direct cell-cell contact of HUVECs with THP-1 cells is a key determinant for amplified CAMs and cytokine expression in the SiO_2_-particle-stimulated cocultures. Subsequently, we also used cell-free supernatants of SiO_2_-particle-activated HUVECs to stimulate THP-1 cells. Similarly, the supernatant of SiO_2_-particle-stimulated HUVECs had no impact on TNF-α or IL-1β production, suggesting that monocyte activation in SiO_2_-particle-stimulated cocultures also requires direct cell-to-cell contact (Figure 8C). In contrast, the supernatant of SiO_2_-particle-stimulated HUVECs was capable of markedly increasing TF expression in THP-1 cells (80.29 ± 2.76%) (Figure 8D); there was no significant difference compared with TF expression in THP-1 cells (82.03 ± 2.56%) in SiO_2_-particle-activated cocultures (Figure 7), indicating that soluble factors from activated ECs may significantly contribute to TF expression in monocytes in cocultures activated with SiO_2_ particles. It was attributed to higher IL-6 and IL-8 levels in the supernatant of SiO_2_-particle-stimulated HUVECs because IL-6 and IL-8 have been reported to induce an increase in the surface expression of TF in monocytes [40]. 

**Figure 9:**
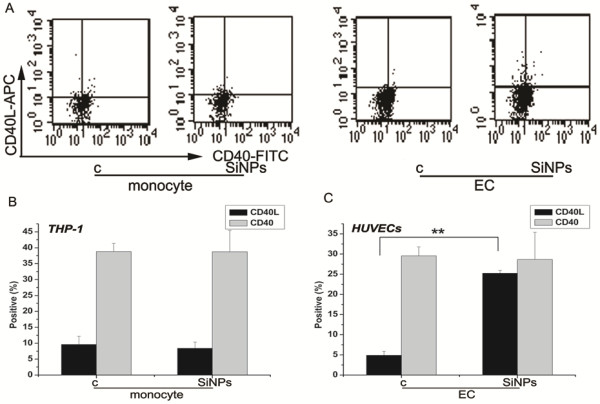
**CD40L and CD40 expression induced by SiO**_**2**_** particles in HUVECs and THP-1 cells. A**): Dot-plot of flow cytometry analysis showing the expressions of CD40L and CD40 on THP-1 cells and HUVECs; **B**): The bar graph shows the percentage of CD40L or CD40 positive THP-1 cells; **C**): The bar graph shows the percentage of CD40L or CD40 positive HUVECs. Untreated HUVECs or THP-1 cells served as controls. Data represent the mean ± SEM, n = 3. **p < 0.01 vs. control. (c: control, SiNPs: SiO_2_ particles, EC: endothelial cells).

In contrast with soluble factors, the role of direct cell-to-cell contact in amplifying biological effects in particle-stimulated coculture systems has received little attention. A previous study has found that supernatants from macrophages strongly induce adhesion molecule expression on ECs [41]. However, our findings suggest that direct cell-to-cell contact is a prerequisite for enhanced cytokine generation (IL-6, IL-8, MCP-1, TNF-α, and IL-1β) and CAMs expression in SiO_2_-particle-stimulated cocultures. In general, upon cell-to-cell contact and ligand-receptor engagement, intracellular signaling is induced in a bidirectional manner. CD40 is a member of the tumor necrosis factor receptor (TNF-R) superfamily that is activated by ligand of CD 40 (CD40L), a member of the TNF-α family. CD40 and CD40L were originally known as co-stimulatory molecules that are indispensable for the function of antigen-presenting cells and activated CD4^+^ T cells. Recent studies found that CD40–CD40L interactions between ECs and monocytes can significantly increase CAMs expression in ECs or IL-1β release from monocytes [12,42]. Thus, to further investigate the possible cell-to-cell contact-dependent signaling mechanism, CD40 and CD40L expression in monocytes and ECs were analyzed after exposure to SiO_2_ particles. As shown in Figure 9, CD40L expression in HUVECs was strongly induced by SiO_2_ particles. In contrast, exposure to SiO_2_ particles had no effect on CD40 expression in HUVECs; similar results were also observed in THP-1 cells exposed to SiO_2_ particles (Figure 9). As reported in a previous study, CD40L synthesis in HUVECs was likely dependent on a redox-sensitive mechanism [13]. 

### Activation of the ROS, MAPK and NF-κB pathways

**Figure 10:**
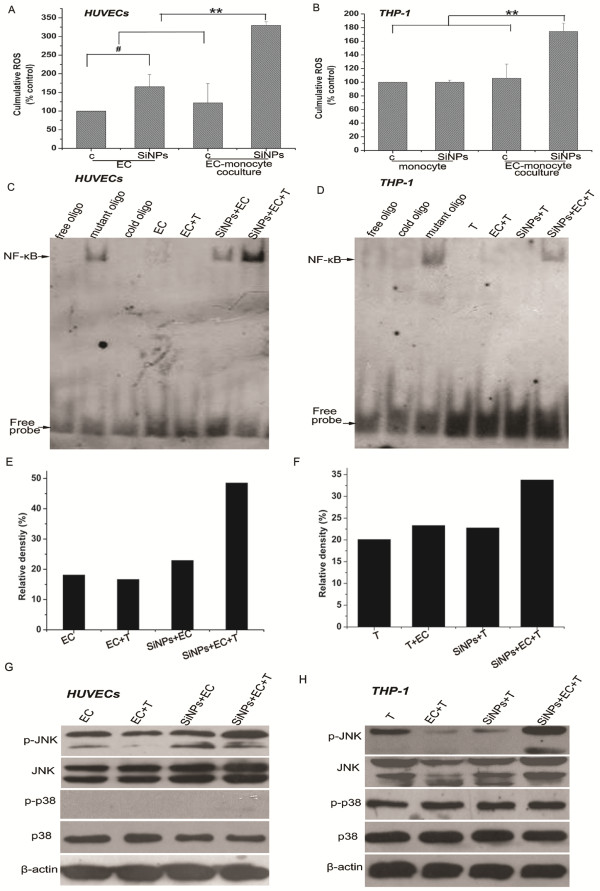
**ROS generation and NF-κB, JNK and p38 activation by SiO**_**2**_** particles in HUVECs and THP-1 cells in mono- and co-cultures. A**, **B**): ROS generation by SiO_2_ particles in HUVECs and THP-1 cells in mono- and co-cultures. **C**, **D**): The activation of NF-κB by SiO_2_ particles in HUVECs and THP-1 cells in mono- and co-cultures. NF-κB DNA-binding activity was assayed by the electrophoretic mobility shift assay (EMSA) as described in the Methods section. The detection of band specificity of NF-κB activation was measured with unlabeled oligo-, cold and mutated NF-κB oligonucleotides. **E**, **F**): The relative density of the bands from EMSA by gray value analysis. **G**, **H**): The activation of JNK and p38 by SiO_2_ particles in HUVECs and THP-1 cells in mono- and co-cultures. Aliquots of the cell lysates were separated by SDS-PAGE and analyzed for protein expression by Western blotting, as described in the Methods section. β-actin was used as an internal control to monitor for equal loading. Data represent the means ± SEM; n = 3. #p < 0.05, **p < 0.01, a significant difference between compared groups. (c: control, SiNPs: SiO_2_ particles, EC: endothelial cells, T: THP-1 cells).

Subsequently, ROS production was monitored by flow cytometry with 2′,7′-dichlorodihydrofluorescein diacetate (DCFH-DA), which is the most widely used probe for detecting intracellular oxidative stress. Moreover, DCFH-DA has also been used as an indicator for the mitochondrial generation of oxidants and peroxynitrite in endothelial cells [43]. As shown in Figure 10, after 24 h, intracellular ROS in HUVECs were significantly induced by SiO_2_ particles; however, in THP-1 cells, SiO_2_ particles failed to increase intracellular ROS levels. Oxidative stress is one of the most important toxicological paradigms and mechanisms for the toxicity of engineered particles [44,45]. Our previous studies have shown that SiO_2_ particles exerted the toxic effects of oxidative stress in HUVECs and PC12 cells [8,46], the current study further found that ROS generation was significantly enhanced or induced by monocyte-endothelial cell interactions (Figures 10A-B). To further clarify the possible signaling pathways underlying SiO_2_-particle-induced monocyte-endothelial cell interactions, the activation of c-Jun NH2-terminal kinase (JNK), p38 MAP kinase and NF-κB was analyzed in THP-1 cells and HUVECs in mono- and co-cultures stimulated with SiO_2_ particles. MAPK represents an intracellular signaling pathway that processes a wide variety of stimuli, including environmental stresses and cytokines, through ERK, JNK and p38 [47]. Among these signaling peptides, p38 MAPK is considered the central regulator of inflammation, and JNK is also involved in inflammatory responses. Furthermore, NF-κB plays a central role in the development of inflammation through the regulation of genes encoding not only pro-inflammatory cytokines but also adhesion molecules, such as E-selectin, VCAM-1 and ICAM-1, chemokines and TF. Our previous study showed that SiO_2_ particles can activate JNK and NF-κB through oxidative stress in HUVECs [8]. The present study further found that the stimulation of cocultures with SiO_2_ particles strongly enhances JNK phosphorylation and NF-κB activation in both HUVECs and THP-1 cells, whereas p38 phosphorylation was not affected by SiO_2_-particles in either the monocultures or cocultures (Figure 10). Previous studies have reported that CD40L-CD40 signaling can stimulate inflammation in an Akt, p38/JNK MAP kinase, and NF-κB dependent manner [12,13]. Thus, it is likely that the enhanced NF-κB and JNK activity in THP-1 cells and HUVECs in SiO_2_-particle-stimulated cocultures is due to CD40-CD40L interactions. 

Taken together, our results indicate that SiO_2_ particles may induce CAMs, chemokines and receptor/ligand expression in ECs resulting in the preferential recruitment of monocytes from blood and their adhesion to ECs. The interaction of monocytes with ECs (via the CD40-CD40L pathway) results in the activation of both ECs and monocytes, which then release more proinflammatory cytokines or chemokines and induce the increased expression of CAMs or TF. Thus, a positive feedback loop may be created that could finally lead to cardiovascular dysfunction.

## Conclusion

In summary, an *in vitro* model system of endothelial cell and monocyte coculture was developed that mimics cell communication within the bloodstream and could lead to a better understanding of the different cellular mechanisms related to the responses after exposure to inorganic particles. Our results indicated that the production of proinflammatory cytokines and chemokines and the expression of CD106, CD62E and TF are significantly enhanced to a greater degree in SiO_2_-particle-activated cocultures than in the individual cell types alone, suggesting that the co-cultures represent a sensitive *in vitro* model system in which to assess the potency of particles and illustrate that the safe application of nanomaterials requires the evaluation of both the direct and indirect proinflammatory and procoagulant potential of particles. Furthermore, our data also demonstrate that SiO_2_ particles can significantly augment proinflammatory and procoagulant responses through CD40–CD40L-mediated monocyte-endothelial cell interactions via the JNK/NF-κB pathway, suggesting that cooperative interactions between particles, ECs, and monocytes may trigger or exacerbate cardiovascular dysfunction and disease, such as atherosclerosis and thrombosis. The findings provide important information that may help guide the future design and use of inorganic particles in biomedical applications.

## Methods

### Preparation and characterization of SiO_2_ particles and Fe_3_O_4_ particles

SiO_2_ [Cat. No: 637246] and Fe_3_O_4_ particles [Cat. No: 637106] were purchased from Sigma-Aldrich (Sigma, St. Louis, MO, USA). The size and shape of these particles were examined under a transmission electron microscope (TEM) (JEOL, Tokyo, Japan). The specific surface area of these samples was determined by the Brunauer– Emmett–Teller (BET) method using a Surface Area Analyzer (ASAP2020, Micromeretics, GA, USA) after pre-preparation of the samples by heating them at 200°C in a stream of N_2_ in excess of 24 h. The hydrodynamic diameter of these particles in endothelial cell medium (ECM) (Sciencell, San Diego, USA) and RPMI 1640 medium (GIBCO, Scotland, UK) was measured with a Malvern Zetasizer instrument (Malvern Instruments, Worcestershire, UK). In the present study, before inoculation into the *in vitro* systems, these particles were sterilized by ethylene oxide, and the amount of residual styrene oxide of the particles was no more than 10 μg/g. A series of particle concentrations ranging from 25 μg/mL to 400 μg/mL was chosen to test the potential effects of the particles on HUVECs or THP-1 cells. The final particle dispersions were prepared freshly before use by serial dilution of the stock suspension (1 mg/mL) in ECM or RPMI 1640 medium (GIBCO, Scotland, UK), followed by intense vortexing. The endotoxin content of the samples was negative at the level of 1 EU/mL.

### Cell preparation and culture

HUVECs were isolated and cultured using a modification of the method described by Jaffe [48]. Briefly, the umbilical vein was rinsed three times with phosphate-buffered saline (PBS) containing 100 U/mL penicillin/streptomycin (GIBCO, Scotland, UK), filled with 0.1% collagenase I (Sigma, St. Louis, MO, USA), and incubated at 37°C for 15 min. Subsequently, the cells were collected by perfusion with PBS and centrifuged at 1,000 rpm for 10 min. After being harvested, the ECs were placed in 75-cm^2^ tissue culture flasks (Corning, US) and grown in ECM. HUVECs between the third and sixth passages were used in our experiments. The phenotype of the ECs was confirmed by performing immunofluorescence with monoclonal antibodies for the von Willebrand factor (Changdao Biotech, China). Human monocytes (THP-1) were purchased from the Cell Bank of Chinese Academy of Sciences (Shanghai, China) and cultured in RPMI 1640 medium with 10% fetal bovine serum (FBS) (BiochromAG, Berlin, Germany) and 100 U/mL penicillin/streptomycin.

For contact coculture of monocytes and HUVECs, 2 mL aliquots of THP-1 cells (1 × 10^6^ cells/well) were added to 6-well plates onto confluent HUVEC layers (5 × 10^5^ cells/well) in ECM. Experiments with contact cocultures were performed in the presence or absence of SiO_2_ particles for 24 h. To determine the role of soluble factors in monocyte-endothelial cell interactions, THP-1 cells (1 × 10^6^ cells/mL) were treated with SiO_2_ particles for 24 h; the cell-free supernatant was then harvested and transferred to stimulate the HUVECs for 24 h. Likewise, HUVECs (5 × 10^5^ cells/mL) were treated with the particles for 24 h, and the cell-free supernatant was transferred to stimulate the THP-1 cells for 24 h. The purity of THP-1 cells or HUVECs separated from cocultures was assessed with cell-specific surface markers (CD11a for monocytes and von willebrand factor (vWF) for HUVECs) using flow cytometry.

### Cell viability assays

To determine the toxicity levels of the particles, cell viability was measured using the MTS method, a type of mitochondrial succinate dehydrogenase assay (Cell Titer 96 Aqueous non-radioactive cell proliferation assay) (Promega, Madison, WI). HUVECs and THP-1 cell cultures were individually prepared at approximately 20,000 cells per well in 96-well plates. The monolayer of HUVECs was approximately 70-80% confluent after 24 h. Serial dilutions of particles (25, 50, 100, 200, and 400 μg/mL) were added, and the cultures were incubated for 24 h. Twenty milliliters of MTS was then added to each well, and the plates were incubated for 4 h at 37°C in an atmosphere of 5% CO_2_ and 100% humidity. The absorbance of formazan was measured at 490 nm using a microplate reader (Labsystems Dragon Wellscan MK3, Finland).

### Particle uptake

To determine the cellular uptake and localization of the particles, HUVECs and THP-1 cells were individually exposed to particles for 24 h and analyzed by electron microscopy. For TEM studies, HUVECs and THP-1 cells were seeded onto a 6-well plate. After incubation for 24 h with particles (100 μg/mL), the excess medium was removed, and the cells were washed with PBS solution, trypsinized and centrifuged. Then, the cell pellets were fixed in a 0.1 M PBS solution containing 2.5% glutaraldehyde for 4 h. The cells were dehydrated through an ethanol series (70% for 15 min, 90% for 15 min, and 100% for 15 min twice) and embedded in Epon Araldite resin (polymerization at 65°C for 15 h). Thin sections containing the cells were placed on the grids and stained for 1 min each with 4% uranyl acetate (in acetone: water, 1:1) and 0.2% Raynolds lead citrate (in water), air dried, and imaged under a transmission electron microscope.

### Intracellular ROS measurement

The production of ROS was measured by flow cytometry using DCFH-DA (Applygen, Beijing, China). Briefly, a 10 mM DCFH-DA stock solution (in methanol) was diluted 4,000-fold in cell culture medium without serum to yield a 2.5 μM working solution. After the exposure of HUVECs or THP-1 cells to SiO_2_ particles (100 μg/mL) in cocultures or monocultures for 24 h, the cells in 6-well plates were washed twice with PBS and incubated in 2 mL of the working solution of DCFH-DA at 37°C in the dark for 30 min. The cells were then washed twice with cold PBS and resuspended in PBS for the analysis of intracellular ROS with a FACScan flow cytometer (Becton Dickinson, San Jose, CA). DCFH fluorescence emission was collected with a 530 nm band-pass filter. The mean fluorescence intensity (MFI) of 10^4^ cells was quantified using Cell Quest software (Becton Dickinson, USA).

### Cytokine measurement

For the analysis of cytokines (IL-6, IL-8, IL-1β, MCP-1 and TNF-α), the supernatants of HUVECs or THP-1 cells in cocultures or monocultures exposed to particles (100 μg/mL) were collected after 24 h, immediately centrifuged to remove the cells, and then frozen at −80°C until the analysis was performed. The amounts of IL-6, IL-8, MCP-1 IL-1β and TNF-α were quantified with an immunoassay kit (R&D Systems, Oxford, UK) according to the manufacturer’s instructions.

### Immunofluorescence flow cytometry

The levels of surface markers expressed on HUVECs and the procoagulant phenotype of THP-1 cells were assessed using flow cytometry. After 24 h of coculture or monoculture in the absence or presence of SiO_2_ particles (100 μg/mL), THP-1 cells were separated and harvested by centrifugation, while HUVEC monolayers, seeded in the 6-well plates, were released from the wells after washing with PBS. The following mouse anti-human monoclonal antibodies were used: ICAM-1 (CD54-PE, eBioscience, San Diego, USA), VCAM-1 (CD106-FITC, BD Biosciences, San Diego, USA), E-selectin (CD62E-APC, BD Biosciences, San Diego, USA), tissue factor (TF) (CD142-PE, BD Biosciences, San Diego, USA), CD11a (Biolegend, San Diego, USA), and vWF ( BD Biosciences, San Diego, USA). In addition, FITC- and APC-conjugated antibodies specific for human CD40 and CD40L (BD Biosciences, San Diego, USA), respectively, were also used to determine the expression of the costimulatory molecules on HUVECs and THP-1 cells. After exposure to particles (100 μg/mL) for 24 h, HUVECs and THP-1 cells were collected and labeled with the above-mentioned specific antibodies at room temperature (RT) for 45 min in the dark, washed extensively, and then subsequently fixed with 1% paraformaldehyde. All samples were analyzed with a BD flow cytometer. The data were analyzed with Cell Quest software.

### Western blot analysis

Total cellular protein extracts were prepared as described in a previous study [49]. Briefly, after a 24-h coculture or monoculture in the absence or presence of SiO_2_ particles (100 μg/mL), as indicated, HUVECs or THP-1 cells were washed once with ice-cold PBS and lysed in ice-cold lysis buffer [50 mM Tris–HCl, 150 mM NaCl, 1% NP-40, 0.1% sodium dodecyl sulfate (SDS), Applygen, Beijing, China] containing 1 mM phenylmethylsulphonyl fluoride (PMSF) (Sigma, St. Louis, MO, USA) and phosphatase inhibitor cocktail (Sigma, St. Louis, MO, USA) for 30 min. After centrifuging the lysates at 12,000 rpm and 4°C for 10 min, the supernatants were collected and stored at −80°C until used. The protein concentrations of these extracts were determined by performing a bicinchoninic acid (BCA) protein assay (Pierce, Rockford, USA). Equal amounts of the lysate proteins (40 μg) were then loaded onto SDS-polyacrylamide gels (10-12% separation gels) and electrophoretically transferred to nitrocellulose (NC) membranes (Amersham Biosciences, US). After blocking with 5% nonfat milk in Tris-buffered saline (TBS) containing 0.05% Tween-20 (TBST) for 1 h at RT, the membrane was respectively incubated with anti-p-p38, p-p-JNK, JNK (1:1,000, rabbit polyclonal antibodies, Bioworld Technology, USA), anti-p-38 (1:1,000, rabbit polyclonal antibodies, CST, USA), β-actin (1:1,000, a mouse polyclonal antibody, Santa Cruz Biotechnology, CA) at 4°C overnight, washed with TBST, and incubated with a horseradish peroxidase-conjugated anti-rabbit IgG/anti-mouse IgG secondary antibody at 37°C for 1 h. The antibody-bound proteins were detected using the ECL chemiluminescence reagent (Millipore, USA).

### Electrophoretic mobility shift assay (EMSA)

The EMSA is classically used to detect the activity of transcription factors and relies upon the principle that DNA bound to protein has decreased mobility through a polyacrylamide gel matrix relative to the corresponding free, unbound DNA. In the present study, the NF-κB activation in HUVECs and THP-1 cells was assessed by EMSA. Briefly, after a 24-h coculture or monoculture in the absence or presence of SiO_2_ particles (100 μg/mL), as indicated, nuclear extracts of HUVECs or THP-1 cells were prepared as described by the instructions for Nuclear and Cytoplasmic Extraction Reagents (Pierce, Rockford, USA). Protein concentrations were quantified by the BCA protein assay. Ten milligrams of nuclear protein was incubated in binding buffer containing 50 ng/μL Poly (dI·dC), 2.5% Glycerol, 0.05% NP-40, 5 mM MgCl_2_ and 20 fmol Biotin end-labeled oligonucleotides at RT for 20 min. The labeled oligonucleotides had the following sequences: 5^′^- AGT TGA GGG GAC TTT CCC AGG C - 3^′^ and 5^′^- GCC TGG GAA AGT CCC CTC AAC T - 3^′^. Cold competition experiments were performed by adding a 100-fold molar excess of unlabeled oligonucleotides. Protein–DNA complexes were separated from the free DNA probe by electrophoresis through 4% native polyacrylamide gels. Gels were dried, and then, the protein–DNA complexes were visualized by the ECL chemiluminescence system.

### Statistical analysis

Data were expressed as the mean ± SD or the mean ± SEM. Statistical comparisons of the means were performed using one-way analysis of variance (ANOVA) with the software SAS 6.12. The differences were considered to be significant when the *p* value was less than 0.05.

### Ethical approval

Ethical approval was given by the independent ethics committee of Shanghai Ninth People’s affiliated to Shanghai JiaoTong University, School of Medicine with the following reference number: 2010–43. Written informed consent was obtained from the patient for publication of this report and any accompanying images.

## Competing interests

The authors declare that they have no competing interests.

## Authors’ contributions

Jiao sun obtained funding and provided financial support for the research project. The study design was constructed by Xin Liu and Jiao Sun. Xin Liu performed the majority of the experiments and data analysis and drafted the manuscript. Yang Xue and Tingting Ding performed part of the cellular experiments. All of the authors have read and approved the final manuscript.
